# Fire in an Icy Desert: Oncolytic Virotherapy for Pancreatic Adenocarcinoma

**DOI:** 10.3390/pharmaceutics18040510

**Published:** 2026-04-20

**Authors:** Alessandra Rossetto, Alberto Reale

**Affiliations:** Department of Molecular Medicine, University of Padua, 35121 Padua, Italy; alessandra.rossetto.1@phd.unipd.it

**Keywords:** pancreatic cancer, oncolytic viruses, immunotherapy, clinical trials

## Abstract

Pancreatic ductal adenocarcinoma (PDAC) remains one of the deadliest malignancies, characterized by early metastasis, dense desmoplastic stroma and a profoundly immunosuppressive, lymphocyte-depleted tumor microenvironment that severely limits the efficacy of current systemic and immunotherapeutic approaches. Oncolytic viruses (OVs), which selectively replicate in and lyse malignant cells while activating antitumor immunity, have emerged as attractive candidates to convert this “cold” tumor into a more inflamed and therapeutically responsive disease. In this review, we summarize clinical evidence on the main OV platforms evaluated in PDAC, including adenovirus, herpes simplex virus, vaccinia virus, parvovirus and reovirus, with a focus on clinical trials. Across these classes of viruses, intratumoral administration has consistently proven feasible and generally well tolerated, with frequent evidence of viral replication, microenvironmental remodeling and immune activation, but only modest and often transient antitumor responses in small, early-phase cohorts. We then discuss key biological and translational challenges that currently limit OV impact in PDAC, such as systemic delivery in the context of pre-existing antiviral immunity and rapid clearance, penetration through the fibrotic stroma, and rational selection of encoded transgenes to reshape myeloid cell-driven, pro-tumoral inflammation and enhance T-cell recruitment. Finally, we outline future directions for the field, including carrier-cell–based systemic delivery, stroma-targeting or cytokine-armed constructs, and combinatorial strategies with chemotherapy and immune checkpoint blockade, arguing that design refinement, innovative combinations and mechanism-driven trial designs will be essential to unlock the full therapeutic potential of oncolytic virotherapy in PDAC.

## 1. Introduction

**Figure 1:**
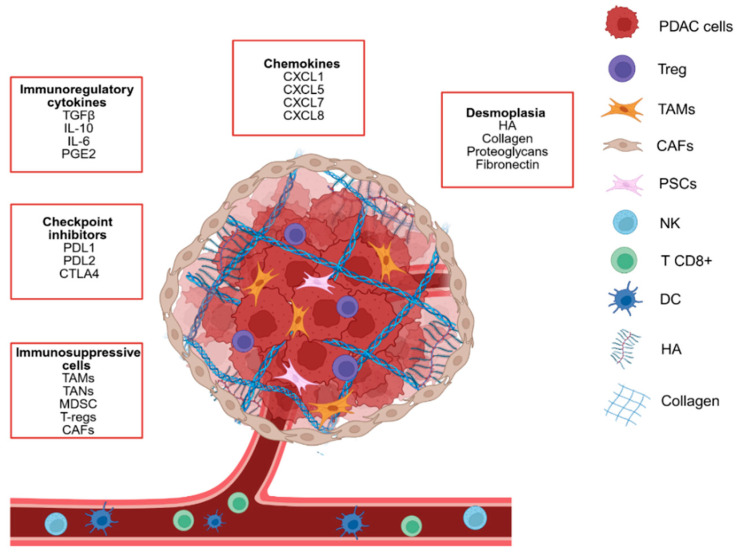
Schematic representation of the PDAC tumor microenvironment. PDAC tumor microenvironment is extremely immunosuppressive, due to its enrichment with immunosuppressive cells, immunoregulatory cytokines and desmoplasia reaction. The most represented cells include cancer cells, cancer associated fibroblasts (CAFs), tumor associated macrophages (TAMs), pancreatic stellate cells (PSCs) and regulatory T cells (T-reg), surrounded by a dense extracellular matrix composed of hyaluronic acid (HA) and collagen. A poor infiltration of lymphocytes T CD8+, dendritic cells (DCs) and natural killer cells (NKs) is observed.

Among solid tumors, pancreatic cancer and specifically pancreatic ductal adenocarcinoma (PDAC) represent one of the major therapeutic challenges for contemporary medicine [1]. Indeed, this neoplasm is often clinically silent during its early stages, but it is biologically aggressive due to both local invasion and metastasis [2]. As a result, PDAC has a very low 5-year survival rate, approximately 13%, a figure that decreases further in patients who are not eligible for surgery [3]. Whereas PDAC has been historically considered as a relatively rare tumor, its incidence is increasing in developed countries, because of the aging population and other, not well-understood factors which might be environmental [4]. PDAC is expected to become the second-leading cause of cancer-related deaths worldwide by 2030 [5]. Several factors contribute to PDAC aggressiveness: First, both early perineural and microvascular invasion lead to the precocious formation of metastases, especially in the liver [6]. Second, it is characterized by desmoplasia, i.e., the deposition around lesions of an extremely dense stroma that occupies more than 70% of the total tumor volume and plays an essential role in PDAC progression. This mechanical barrier represents the main obstacle to chemotherapeutic drug delivery, causing an intratumoral fluid pressure increase that promotes vascular compression, a reduction of tissue perfusion and a hypoxic microenvironment [6]. Additionally, PDAC is immunologically “cold”, due to the consistent infiltration of suppressive cells (myeloid and regulatory cells), absence of CD8+ T cells and NK cells and presence of immunosuppressive molecules (Figure 1) [6].

Among the emerging therapeutic options, a highly promising one is represented by oncolytic viruses (OVs) [7].

While it has been known for decades that some viruses replicate better in malignant rather than in benign cells, in recent years, advancements in genetic engineering techniques have allowed for a more precise control over viral replication and the targeted expression of therapeutic genes [8]. Furthermore, OVs are integrated into the evolving and promising landscape of cancer immunotherapy, because they serve as immunogenic stimuli in the otherwise immunosuppressive tumor microenvironment (TME). Several mechanisms are potentially involved, including fostering of immunogenic cell death (ICD) with release of pathogen-associated molecular patterns (PAMPs) and damage-associated molecular patterns (DAMPs), and increased recruitment of cells that mediate adaptive cell immunity [9]. Therefore, it has been hypothesized that OVs can synergize with other immunotherapeutic strategies, such as immune checkpoint inhibitors or CAR-T cells, mainly because viruses can interfere with different immunosuppressive mechanisms [10].

In this review, we summarize the available literature on the most studied OV platforms for the treatment of PDAC, focusing on results from clinical trials.

## 2. Main Oncolytic Virus Classes in Clinical Trials for the Treatment of PDAC

As mentioned above, oncolytic viruses (OV) are naturally occurring or genetically modified viruses that specifically target and destroy cancer cells, while minimizing damage to normal healthy cells [9].

This selectivity is the result of different factors, including the impaired function of intrinsic cellular antiviral pathways in cancer cells, their higher rate of replication and their expression of specific viral receptors [11].

OVs can be classified in two groups, based on their genome: (i) DNA viruses, including adenoviruses, vaccinia viruses and herpes simplex viruses (HSV); (ii) RNA viruses such as reoviruses, coxsackieviruses, measles virus and Newcastle disease virus [12]. The most common types of OV are single-stranded RNA and double-stranded DNA viruses: The first ones usually have a smaller genome compared to DNA viruses, limiting their cargo capability and are characterized by higher genetic instability. DNA viruses, instead, have larger encoding ability and the rate of genomic mutations is lower [8].

OVs can explicate their anti-tumor action through two different mechanisms: direct oncolysis of target cancer cells, inducing their death, and activation of both innate and adaptive host immune system, due to the release of Pathogens Associated Molecular Patterns (PAMPs), Damage Associated Molecular Patterns (DAMPs) and Tumor-Associated Antigens (TAAs) that promote inflammation and an enhanced immune response. In this way, as mentioned above, they are able to transform “cold” tumors into “hot” tumors, acting as immunotherapeutic agents that trigger the immune response against cancer cells [13].

Several oncolytic viruses have been approved for the treatment of solid tumors: First, Rigvir, a Picornavirus, was approved in Latvia for melanoma therapy. Second, the adenovirus H101 was accepted in China for head and neck cancer treatment. In 2015, Talimogene Laherparepvec, based on Herpes Simplex Virus type 1, was approved in the USA and the European Union for the treatment of non-resectable melanoma. Finally, in 2021 another HSV-1-based oncolytic virus (named DELYTACT) was approved in Japan for the treatment of glioblastoma [14].

Despite these advancements in virotherapy, targeting PDAC is still an issue.

Here we aim to focus on the main oncolytic viruses that have been analyzed in patients affected by PDAC (Table 1).

### 2.1. Adenovirus

Adenoviruses (AdVs) are non-enveloped icosahedral viruses, characterized by a linear dsDNA genome ranging in size from 24 to 40 kbp. Their replicative cycle relies essentially on two proteins, E1A and E1B, which block the function of the pRb and p53 proteins, respectively, promoting cell cycle progression, DNA synthesis and apoptosis inhibition. Oncolytic adenoviruses (oAdVs) often achieve tumor selectivity through specific deletions of these E1A/E1B regions, exploiting the defective antitumor suppressor pathways in cancer cells to enable replication and selective lysis [15].

AdVs are among the most studied viruses in the field of oncolytic virotherapy, due to their broad tropism, low pathogenicity in humans, well-studied structure and replicative mechanisms, simplicity of production and limited recombination events. However, despite low direct pathogenicity in most cases, clinical experience with non-replicative adenoviral vectors has shown that some patients can develop very rare but life-threatening autoimmune reactions, prompting caution in the therapeutic use of adenoviruses [15].

There are several clinical trials that focus on AdVs for the treatment of PDAC.

The first oncolytic adenovirus trial for PDAC began almost 20 years ago, with the administration of ONYX-015 (dl1520), an E1B-55kD gene-deleted adenovirus that selectively replicates in p53-mutant cancer cells, to patients with unresectable pancreatic cancer. This Phase I dose escalation clinical study, based on CT-guided injection or intraoperative injection, showed ONYX-015 had a good safety profile (91% of patients reported grade 1 symptoms and their duration was short) but no viral particles were detected in patients in the days following the treatment [16].

Two years later, another clinical study analyzed the application of ONYX-015, alone or in combination with gemcitabine, in patients with advanced pancreatic adenocarcinoma or with metastatic disease, employing endoscopic ultrasound-guided injection as a delivery system. The therapy proved to be well tolerated and caused partial tumor regression in two patients [17].

In 2020, an innovative armed oncolytic adenovirus, Ad5-yCD/mutTK(SR39)rep-ADP (Ad5-DS), was generated, through the insertion of two genes involved in increasing its anti-tumoral activity (sensitization of tumor cells to chemotherapy/radiotherapy and oncolytic augmentation). In particular, it expresses the mutant (SR39) herpes simplex virus thymidine kinase fusion/yCD/mutTKSR39 gene and ADP genes regulated by the immediate early human cytomegalovirus promoter. Clinical tests (NCT02894944) performed on nine patients with locally advanced pancreatic cancer, highlighted the safety and the potential of Ad5-DS, locally administered, in combination with gemcitabine, delivered systemically, for PDAC treatment. Indeed, no additional toxic effects were observed in any of the three treatment cohorts, except fever in two patients during the initial stages. Moreover, eight participants showed stable disease at the first restaging (12 weeks) with a disease control rate of 100%. However, although eight patients received stereotactic body radiation therapy after gemcitabine administration, none of them had a sufficient response to proceed with surgical resection [18].

Following these promising results and due to the progressive improvements in molecular biology and molecular virology techniques, other clinical trials focused on using engineered anti-cancer adenoviruses. In 2021, a Phase I study (11,260) analyzed the effect of Ad5-yCD/mutTK(SR39)rep-hIL-12, a replicative adenovirus expressing yeast cytidine deaminase/mutant SR39 thymidine kinase (yCD/mutTK(SR39)) and the immunostimulatory cytokine human IL-12 (hIL12), in 12 subjects with metastatic pancreatic cancer. Like other clinical trials using similar oncolytic adenovirus agents, over 90% of adverse events observed were mild (grade 1) or moderate (grade 2) and did not require medical intervention to be resolved, with few exceptions including rare instances of hypotension, syncope, hyperglycemia or lymphopenia. Additionally, the immune system activation and the increase in the median survival duration suggest an Ad5-yCD/mutTK(SR39)rep-hIL-12 potential immunostimulatory and antitumoral profile [19].

Among the armed oncolytic adenoviruses used in clinical trials, VCN-01 emerged, a virus designed to replicate in cancer cells with defects in the RB1 pathway. VCN-01 expresses the human sperm hyaluronidase PH20, an enzyme that helps disrupt the characteristic desmoplastic stroma surrounding cancer cells. Eight patients with unresectable advanced PDAC were treated intratumorally with VCN-01 in combination with standard doses/schedules of either gemcitabine or nab-paclitaxel plus gemcitabine (NCT02045589). While the virus replicated efficiently in the tumor mass, expressed PH20 to promote stroma destruction and was well-tolerated in the most of patients, with limited adverse events including asthenia, fever and transaminase increases, one patient developed a pancreatic fistula with intraabdominal fluid collection, leading to a fatal gastrointestinal hemorrhage. The association of this fatal adverse event with oncolytic virotherapy is uncertain. Unfortunately, in all patients the disease eventually progressed because of the onset of new pancreatic or metastatic lesions [20].

Thereafter, Garcia-Carbonero et al., in a Phase IIb clinical study (NCT02045602), treated 12 and 14 patients with metastatic PDAC with gemcitabine and nab-paclitaxel in combination with VCN-01 intravenously. The therapy resulted in an improvement of the overall survival and an increased duration of response compared to patients in the control group treated with chemotherapeutic agents alone, demonstrating the potential efficacy of the virus. Moreover, deeper molecular analysis highlighted the capability of the virus to remodel the tumor microenvironment. In particular, in VCN-01 treated patients, the activation of an inflammatory immune response was confirmed. Specifically, an increase in CD8+ T cells, a decrease in Treg cells, an upregulation of the programmed cell death protein 1/programmed cell death ligand 1 axis and CTLA-4 signaling was observed in tumor biopsies. Additionally, this study showed VCN-01 safety when delivered systemically; most of the treated patients developed mild adverse events (fever, vomiting, asthenia, nausea and alanine aminotransferase increase were the most frequent), even if some more severe events occurred (thrombocytopenia, febrile neutropenia, neutropenia and dose limiting toxicity) [21].

Due to these previous encouraging results, TherivaTM Biologics announced the design of a novel, multinational Phase 3 clinical trial of the aforementioned oncolytic agent VCN-01 in combination with gemcitabine/nab-paclitaxel chemotherapy for the first-line treatment of metastatic PDAC.

Additionally, the Baylor College of Medicine (BCM) and Baylor St. Luke’s Medical Center (BSLMC) are setting up a Phase I/IIa clinical trial to test the safety and antitumoral activity of LoAd703, an oncolytic adenovirus armed to express two immune system stimulator molecules, TMZ-CD40L and 4-1BBL. The oAdV will be delivered intratumorally, through ultrasound-guided percutaneous injection, in combination with gemcitabine/nab-paclitaxel, with or without the monoclonal anti-PD-L1 antibody atezolizumab.

### 2.2. Herpes Simplex Virus

Herpes Simplex Virus type 1 (HSV-1) and Herpes Simplex Virus type 2 (HSV-2), belonging to the Herpesviridae family, are double-stranded DNA viruses, structurally composed by four distinct elements, i.e., envelope, tegument, icosahedral capsid and central core. Their 150 kbp genome, once released in the nucleus of permissive host cells, is efficiently transcribed leading to productive virus replication [22].

HSV-1 and HSV-2 show many characteristics that make them interesting platforms for the development of oncolytic viruses. First, they hold 30 kbp of DNA which is not strictly necessary for their replication; therefore, the viruses can accommodate multiple transgene insertions. Second, the absence of viral genome integration inside the host genome reduces the insertional mutagenesis events. Moreover, HSV is the only OV platform which ensures that an unexpected pathogenic infection could be controlled by well-established antiviral treatments, such as acyclovir. Finally, HSV viruses have broad tropism that permits their use in different solid cancers, including glioblastoma or pancreatic ductal adenocarcinoma [22].

The first clinical trial using HSV-1 as an oncolytic virus started in 2005. HF-10, a naturally mutated virus derived from the parent virus strain HSV-1 HF (which causes enhanced formation of syncytia), was examined for the first time as a single therapy to treat six PDAC patients with non-resectable disease in a Phase I clinical trial. The viral treatment, delivered intratumorally during laparotomy, was completely safe (no adverse events detected), immunostimulatory (increased infiltration of CD4+ and CD8+ T cells being observed) and in some patients, it prolonged the overall survival compared to ordinary bypass intervention [23].

Other clinical trials have started in recent years and are ongoing.

In 2017 a Phase I, open-label, multi-center study (NCT03252808) began, focusing on the recommended dose of TBI-1401 (HF10) injected intratumorally, in combination with chemotherapy (gemcitabine + nab-paclitaxel or TS-1), in patients with stage III or IV unresectable pancreatic cancer. No further information is currently available on this trial.

In 2021, a Phase IIb/II clinical trial (NCT04637698) started the study of safety and efficacy of OH2 in patients with locally advanced or metastatic pancreatic cancer who had failed first-line standard treatment. OH2 is an oncolytic virus derived from the HG52 HSV-2 strain that was specifically engineered to guarantee increased selectivity and to encode the human granulocyte macrophage colony-stimulating factor (GM-CSF) gene to elicit a more potent antitumor immune response. This trial is based on intratumoral virus delivery, with repeated administrations. Currently, no results are available.

In 2022, a multicenter, open, single-arm design clinical trial (NCT05162118) analyzed VG161, an oncolytic HSV-1 genetically engineered in order to express three immunostimulatory genes (human IL-12, human IL-15 and PDL1B). In particular, HSV-seropositive patients with advanced pancreatic cancer (metastatic PDAC) were treated with VG161 in combination with nivolumab injection (PD-1 inhibitor), to explore the safety, the tolerability, the recommended safe dose and the preliminary efficacy of the combinatory treatment. Even though no specific published data are available, some preliminary results were presented in March 2023 at the 38th Annual Meeting of the Society for Immunotherapy of Cancer (SITC). Indeed, the virus was found to be safe because of the absence of dose limiting toxicities and limited adverse events (except for one case with a related cytokine release syndrome). Moreover, remodeling of the tumor microenvironment inside the tumor mass was observed: according to the results of single cell data analysis, the number of cancer cells was reduced, while there was a significant increase in T and NK cell infiltration, demonstrating VG161 capability of transforming the “cold” PDAC to a “hot” cancer. Moreover, patients treated with the OV were also more sensitive to subsequent immune checkpoint inhibitor therapy (nivolumab). Overall, these data indicate the potential of a future use of VG161 in PDAC treatment [24].

Another HSV-1 based oncolytic virus that was tested in nine patients with treatment-refractory advanced PDAC was talimogene laherparepvec (T-VEC), the only FDA-oncolytic virus approved, specifically for the treatment of melanoma. T-VEC was safe after endoscopy-guided intratumoral injections, with manageable adverse events (no serious toxicities were directly linked to its administration). Moreover, it seemed to be involved in stabilizing the disease, although no regressions of the disease were observed. It is worth mentioning that GM-CSF, the therapeutic gene encoded by T-VEC and other OVs, might not be an optimal choice for the treatment of PDAC, since this cytokine is apparently involved in immunosuppression and cancer progression [25,26].

Overall, these findings highlight the potential of HSV oncolytic viral therapies, in combination with other treatments, in PDAC. As a main drawback, widespread pre-existing immunity to HSV-1 exists in the general population, which imposes a limitation on the systemic administration of the virus due to neutralizing antibodies. In the TME on the other hand, the presence of pre-existing immunity could paradoxically be beneficial and enhance the therapeutic effect, as it has been recently shown in a different tumor (glioblastoma) which is also very resistant to immunotherapy [27].

### 2.3. Vaccinia Virus

Vaccinia Virus (VACV) is a large, enveloped, double-stranded DNA orthopoxvirus, originally used as a vaccine against smallpox. The most widely used experimental strains include Lister, Western Reserve (WR), Wyeth, Copenhagen, Ankara and Tiantan strains, which can be either non-replicative or replication competent [28].

VACV presents different advantages in oncolytic virotherapy: First, it replicates exclusively in the cytoplasm, preventing insertional mutagenesis. Second, it holds a large genome (190 kbp), permitting the insertion and the expression of multiple therapeutic transgenes. Moreover, it has a rapid and lytic replication cycle and can produce viral progeny in hypoxic environments. Finally, it presents broad tropism because it does not have known limitations on specific receptors during its entry [28].

Currently, different clinical trials testing VACV are ongoing, but no preliminary data are accessible.

In 2025, a new Phase IIa clinical trial (NCT07006077) evaluated the efficacy of intratumorally injection of hV01, an oncolytic vaccinia virus expressing human IL-21 cytokine, in patients with advanced pancreatic cancer.

The same virus (hV01) was analyzed in June 2025, alone or in combination with the PD-1 inhibitor tislelizumab in patients with advanced solid tumors, to determine the safety and the tolerability of hV01 administration at two different frequencies and the effect of association with the aforementioned monoclonal antibody (NCT07185243). No results are available, as these trials have not been completed yet.

### 2.4. Parvovirus

Parvoviruses are small, non-enveloped, icosahedral viruses characterized by a linear directional single-strand DNA (ssDNA) genome comprising, ca., 5 kbp [29].

Among the different species of Parvovirus, Rodent protoparvovirus 1 has been investigated for the development of an oncolytic virus, as it is not associated with any disease in humans and there is no expected pre-existing antiviral immunity in the human population. Furthermore, parvoviruses display intrinsic oncolytic and oncosuppressive properties and they could activate a robust anticancer immune response [30]. Drawbacks of oncolytic parvoviruses include their very small genome size, which does not allow the insertion of any therapeutic gene. Furthermore, parvoviruses are completely restricted to actively replicating cells. While this could seem an attractive feature for an oncolytic virus, counterintuitively, it can become a disadvantage because some malignant cells in the TME, especially cancer stem cells, are not always undergoing replication [30].

A vector based on Rodent protoparvovirus 1, ParvOryx (H1-PV) has been used in a clinical trial; despite being a rodent virus, it is also able to infect and replicate in some human cancer cells. Based on previous favorable results obtained in pre-clinical studies, a clinical trial (ParvOryx02; NCT02653313) started, involving seven patients with PDAC showing at least one hepatic metastasis and being refractory to first line therapy [31]. The preliminary results highlight the excellent tolerability of the virus, with no dose-limiting toxicities events. Moreover, there was some evidence of effectiveness: one patient presented a partial response to virotherapy, showing increased survival and in all subjects an immune activation was observed, suggesting ParvOryx is able to induce immunomodulation after its administration [32].

### 2.5. Reovirus

Reoviruses are non-enveloped viruses with a segmented, double-stranded RNA genome, belonging to the *Reoviridae* family; they are promising oncolytic agents, due to their natural ability to selectively target and kill cancer cells. Type 3 Dearing (T3D), the most widely studied strain, was used for the development of Pelareorep (REOLYSIN^®^), an oncolytic virus, without genetic engineering. It is non-pathogenic in humans, and it has characteristics, such as cell carriage, targeted delivery and capability of triggering host immune response, that make it appealing as a tool in cancer therapy [33].

In 2009, pelareorep was tested in combination with gemcitabine in 34 patients with advanced PDAC in a Phase IIb clinical trial (NCT00998322). Overall, this treatment was well tolerated, with manageable and self-limited toxicities. One partial response, 23 stable diseases and five cases of progressive disease were observed. A deeper analysis revealed the presence of viral replication inside the tumor mass, demonstrating the ability of pelareorep to cross the desmoplastic stroma and reach the tumor microenvironment. Finally, viral presence was associated with an increase in apoptosis events, suggesting that the combination of virus and gemcitabine could act as a pro-apoptotic factor against PDAC [34].

The following year, a randomized Phase 2 study was started in chemotherapy-free patients with metastatic pancreatic adenocarcinoma, in combination with carboplatin and paclitaxel. Between the two groups (Arm A = 36 patients treated with combinatory therapy and Arm B = 37 patients treated with chemotherapeutic drugs alone), no difference in progression-free survival was observed, even if the virus was found to be safe to administer and well tolerated. Moreover, the presence of K-Ras mutations, associated in the literature with an increased cytopathic effect of the virus, seemingly did not affect the efficiency of pelareorep but could promote a higher recruitment of immunosuppressive cells in the tumor mass [35].

In 2015, a Phase 1b single arm (NCT02620423) study involving 11 PDAC patients previously subjected to first-line treatment started, combining pelareorep with chemotherapy (5-fluorouracil, gemcitabine or irinotecan) and immunotherapy (pembrolizumab, an anti-PD-L1 antibody). The treatment was found to be safe, with mostly grade 1 or grade 2 treatment-related adverse events, highlighting the possibility to combine pelareorep and pembrolizumab as chemotherapeutic agents. Moreover, there were some hints of efficacy, with disease control and long-term benefits achieved in three patients, stable disease for some months in two subjects and partial response in one patient. A molecular analysis of tumor biopsies also revealed the expression of viral genes inside the tumor mass, demonstrating virus ability to replicate in the tumor microenvironment and the generation of new T-cell clones in peripheral blood, suggesting the activation of the host immune system against cancer cells [36].

To establish an innovative, chemotherapy-free regimen, the combination of pelareorep and pembrolizumab alone was evaluated in a Phase II clinical trial involving 13 patients with metastatic PDAC that progressed after first-line treatment (NCT03723915).

As in the previous study, this immunotherapeutic treatment was found to be safe, with limited (grade 1 or 2) adverse events occurring in the subjects. However, the anti-tumoral efficiency was modest, with no complete remission, 1 partial remission, 4 stable disease and 7 progressive disease (non-responders) indicating poor potency in unselected PDAC patients [37].

## 3. Conclusions and Future Directions

As we described above, multiple classes of OVs have been tested for the treatment of PDAC, with several of them reaching the clinical trial stage [38]. OVs are particularly interesting and promising against this neoplasm for which current immunotherapeutic strategies are encountering major problems, due to both immune exclusion and the highly immunosuppressive tumor microenvironment. As a result, ICIs are not effective because of the scant presence of T cells to activate, on the other hand, CAR-T cells do not readily infiltrate the TME or become exhausted [39]. Despite their promise, several issues need to be addressed before OVs become a real clinical therapeutic option for PDAC. While clinical trials have demonstrated outstanding safety and hints of remodeling of the tumor microenvironment and therapeutic efficacy, a significant extension of the life expectancy of patients has not been observed, also because of the small number of participants [40]. In the following section, we consider the most important challenges that, in our opinion, OVs will face in their development as an effective treatment for PDAC.

### 3.1. Systemic Delivery

PDAC is a disease that is characterized by an early metastatic process, which contributes decisively to its very poor prognosis. The most affected sites are the liver, the peritoneum, the lungs and less commonly, bones, adrenal glands or other organs [41]. Approximately 50% of PDAC patients have distant metastases upon diagnosis [42].

Therefore, effective treatment for this disease should ideally be administered systemically to target both primary and secondary tumors. However, most OVs that have been tested in clinical trials have been delivered intratumorally (Table 1), relying on an immunologic abscopal effect to achieve systemic efficacy, while it is known that the antigen landscape of metastasis can differ significantly from that of the primary malignancy.

The main barriers to the systemic delivery of OVs are neutralization by antibodies (which is especially prominent in the case of viruses with high seroprevalence in the population), phagocytosis by immune cells especially in the spleen and in the liver, and non-specific uptake by other tissues in which the virus does not cause damage [43]. Even if in some cases, viruses with low seroprevalence can be delivered intravenously, the subsequent presence of neutralizing antibodies is a possible obstacle to repeated dosing. Indeed, there are contrasting reports on this topic. For example, a study regarding reoviruses found that the presence of antibodies enhanced delivery to the tumor by favoring the uptake by mononuclear cells which, afterwards, traveled to the cancer site [44]. However, the immune response in the bloodstream causes confusion and variability in the systemic bioavailability of OVs, with outcomes that might also depend on individual, patient-specific factors. A recent study documented that IgM antibodies in dyslipidemic patients could enhance the systemic delivery of an oncolytic adenovirus [45]. All these factors are further complicating the interpretation of results from clinical trials.

Therefore, several ideas have been devised to achieve reliable and reproducible delivery of OVs upon systemic administration.

Some viruses, such as adenoviruses, can be encapsulated in polymers or nanoparticles to be shielded from antibodies in the circulation [46]. An interesting alternative lies in the use of carrier cells, which can transport viruses into the TME after being infected ex vivo. An important conceptual advantage of carrier cells is that if they show a tropism for the TME, they can actively migrate towards it, rather than relying on passive diffusion. Furthermore, they could be able to concentrate viral vectors at the tumor site [47]. Several types of carrier cells have been considered for OVs, including mesenchymal stromal cells (MSCs) and different types of leukocytes, such as lymphocytes or monocytes/macrophages [47]. Even though few studies have adopted this approach for PDAC [48], in this specific case it would be especially useful to use carrier cells with a natural capability to infiltrate the desmoplastic stroma of the tumor.

Hybrid approaches have also been pursued, such as coating OVs with extracellular vesicles or embedding OVs in nanoparticles that target leukocytes as carrier cells in vivo [49]. However, it is worth mentioning that unlike any form of coating, living cells can actively move towards tumors even crossing barriers in the presence of appropriate stimuli.

### 3.2. Modification of the Suppressive Microenvironment

**Figure 2:**
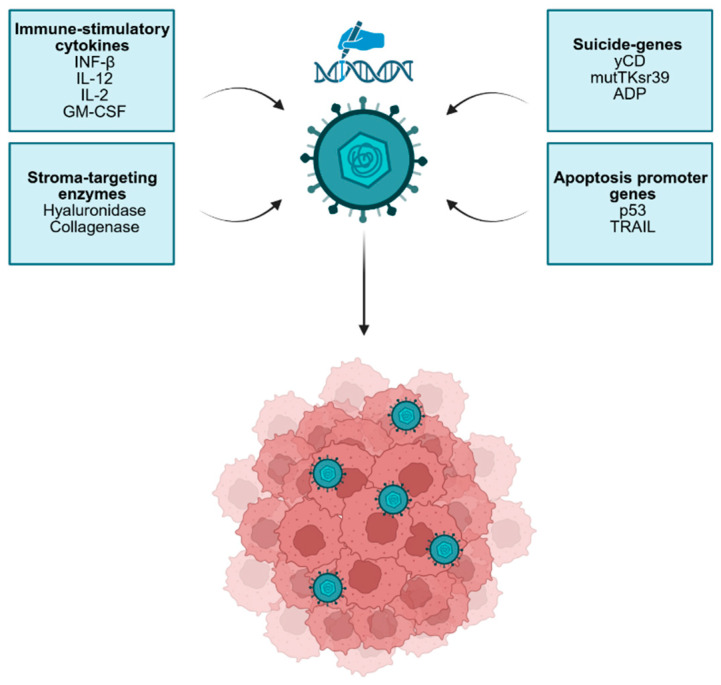
Genetic engineering of OVs for PDAC targeting. Several classes of therapeutic genes that can be integrated into the genome of OVs are highlighted.

The immunology of the TME is fascinating but complicated and the same proinflammatory factors or cells can have an antitumoral or a protumor effect, depending on the context, while the balance can change according to the specific type or subtype of tumor [50]. As a result, the same encoded cytokines might have different effects depending on the specific malignancy that is being targeted [50]. Therefore, in the immunosuppressive and lymphocyte-depleted TME of PDAC, it is likely that an effective OV should enhance the recruitment of T cells, alter the function of immunosuppressive myeloid cells and possibly remodel the stroma, by killing cancer-associated fibroblasts, affecting their capacity to produce extracellular matrix or both (Figure 2) [38,51].

As mentioned above, enzymes that degrade the extracellular matrix have been included in OVs as therapeutic genes, demonstrating the possibility for OV to remove the desmoplastic barrier, promoting both drug and immune system infiltration [52,53].

Among enzymes that promote TME remodeling, hyaluronidase stands out due to the central role that hyaluronic acid (HA) plays in PDAC proliferation, invasiveness, and treatment resistance [54]. Although the concept of depleting stromal HA has been previously proposed and evaluated in several clinical trials, these studies have generally failed to demonstrate an increased therapeutic effect [55]. In contrast, the combination of oncolytic viruses with hyaluronidase remains under active investigation and has shown promising results.

VCN 01, the first oncolytic virus engineered to express a hyaluronidase (PH20), has been tested in both preclinical models (NP 9 and TP 11) and in PDAC patients. In these settings, VCN 01 induces degradation of the HA enriched tumor stroma, leading to a reduction in tumor volume and tumor stiffness [52].

More recently, OVV Hyal1 has emerged as a novel recombinant vaccinia virus encoding a soluble version of the Hyal1 hyaluronidase. Administration of this virus has been associated with enhanced antitumor efficacy in several subcutaneous mouse tumor models compared with its negative control, resulting in improved intratumoral dissemination of chemotherapeutic agents and activation of the immune system [52]. Overall, these findings suggest that local, in situ expression of the enzyme—such as that driven by the virus—increases its functional efficiency [56].

Moreover, in PDAC, a pro-tumoral type of inflammation is usually active, promoting tumor growth and a dysfunctional, ineffective immune response; this form of inflammation is also a very interesting target for therapeutic genes [57].

The tumor microenvironment (TME) of PDAC is profoundly immunosuppressive, characterized by extensive desmoplastic reaction, hypoxia, abundant immunosuppressive cells, and paucity or dysfunction of immune effector cells, making PDAC one of the most challenging cancers to treat [56].

The immunologically “cold” nature of this microenvironment renders PDAC notably resistant to immune checkpoint inhibitors (ICIs) as a monotherapy, with objective response rates typically below 2% [58].

Despite their intrinsic immunostimulatory activity, which stems from their lytic replication in tumor cells, the therapeutic potential of oncolytic viruses is frequently enhanced by “arming” them with multiple transgenes. These modifications increase the secretion of immunostimulatory molecules, improve the recruitment of effector immune cells, and help counteract local immunosuppressive mechanisms [59].

One of the most extensively studied targets is IL-12, owing to its central role in both adaptive and innate immune responses. Its main effects include the induction of IFN γ production, activation of cytotoxic T lymphocytes, and promotion of Th1 cell differentiation [60]. MeVac FmIL-12 is the first oncolytic virus encoding hIL 12 tested in preclinical PDAC models. This virus, which expresses an IL-12 fusion protein, induced complete remissions in 90% of mice, while promoting activation of both Th1 and CD8+ T cell responses and upregulating multiple effector cytokines, including IFN γ and TNF α [61].

Similar results were obtained with VSVΔM51, a recombinant oncolytic vesicular stomatitis virus engineered to express IL-15. In particular, the combination of this OV with NKT cell activation therapy prolonged survival more effectively than NKT cell therapy in combination with the VSV construct lacking the therapeutic transgene. Furthermore, the improved tumor control was associated with increased immune cell infiltration and enhanced antitumor effector functions [62].

Among cytokines, another important target for immunotherapy is the OX40-OX40L axis, central to modulate the T cells response [63]. mOX40L-armed HSV 1 has emerged for its ability to remodel the PDAC TME in KPC mouse models, impacting negatively PDAC development. Specifically, it activates the tumor infiltrating CD4^+^ T cell response, mitigates cytotoxic T lymphocyte exhaustion, reduces the frequency of regulatory T cells (Tregs), and reprograms cancer associated fibroblasts (CAFs), macrophages, and neutrophils toward a proinflammatory phenotype, resulting in delayed tumor growth [64].

In addition to TME remodeling and immune system modulation, OVs can be used as platforms for suicide gene therapy, an approach in which the virus expresses a non-human gene encoding a prodrug converting enzyme that is preferentially produced by cancer cells, thereby enabling localized conversion of the prodrug into a cytotoxic compound. Of the several suicide gene systems identified, the cytosine deaminase/5-fluorocytosine and the herpes simplex virus thymidine kinase/ganciclovir are the most diffused [65].

Despite the limited number of in vivo studies evaluating combination therapy between oncovirotherapy and suicide gene systems, the available data appear promising. Of particular interest is the development of an oncolytic measles virus encoding yeast cytosine deaminase (yCD), which delayed tumor growth and prolonged survival in xenograft bearing mice [66].

In conclusion, oncolytic viruses (OVs) can deliver apoptosis promoter genes (as p53 and TRAIL), thereby inducing apoptosis selectively in cancer cells. Among these molecules, p53 is frequently targeted due to its central role in the apoptotic process [67]. The activation of p53 mediated apoptosis, induced by the telomerase specific oncolytic adenovirus OBP 702 in a syngeneic tumor model, is associated with increased release of damage associated molecular patterns (DAMPs), suggesting that this oncolytic adenovirus enhances immunogenic cell death and improves the efficacy of PD 1 blockade therapy against PDAC [68].

Taken together, these strategies underscore the potential of oncolytic virotherapy to reprogram the PDAC tumor microenvironment. Rather than considering these factors in isolation, an integrated approach is required in order to simultaneously target multiple components through the depletion of the stromal matrix, the activation of antitumor immunity, and the direct killing of tumor cells.

### 3.3. Combination Therapies

Oncolytic virotherapy represents a promising emerging treatment for numerous solid tumors, including pancreatic ductal adenocarcinoma (PDAC), by exploiting oncolytic viruses’ (OVs) ability to convert a “cold” tumor microenvironment (TME) into a “hot” one [38]. Despite their potential, effective responses to OV therapy remain highly patient-specific, owing to a combination of factors—including cellular pathways that regulate immunological, metabolic, and proliferative signaling—which exacerbate PDAC heterogeneity [69]. Notably, cross-resistance between OV therapy and standard treatments (radiotherapy and chemotherapy) is uncommon, thereby paving the way for combinational approaches [70].

As already mentioned above, OVs have been tested in combination with other therapeutics, especially in clinical trials. A pivotal question is going to be, what are the most effective combinations for all oncolytic virotherapy, including those aimed at PDAC [59].

A “classical” combination, also employed in some of the clinical studies we mentioned, is with chemotherapy, especially the combination with drugs representing the gold standard for PDAC clinical treatment [56,71].

As reported in preliminary in vitro and in vivo studies [72], OV-chemotherapy combinations promote a synergistic antitumor effect, resulting in enhanced pancreatic cancer growth inhibition and improved survival. This novel therapeutic approach holds promise for PDAC patients who cannot tolerate existing chemotherapy regimens.

This strategy must be pursued with care, keeping in mind that oncolytic virotherapy is a form of immunotherapy, therefore requiring consideration of the effect of the selected chemotherapy on the immune system. Some forms of chemotherapy, such as the use of platinum compounds, are immunosuppressive and therefore they could diminish the immunotherapeutic effect while potentiating the cytotoxic effects. The dosing of some chemotherapeutic compounds is also fundamental to boost immunogenic cell death and preferentially eliminate immunosuppressive cells in the TME [73].

Radiotherapy represents another therapeutic modality under investigation for PDAC; notwithstanding, its clinical implementation remains contentious and is predominantly restricted to the management of localized disease, attributable to discordant evidence regarding survival outcomes [74]. At present, empirical data on the integration of oncolytic viruses (OVs) with radiotherapy are limited, with scant findings derived from in vivo models; nonetheless, the extant results substantiate that such combinatorial regimens markedly augment tumor cell apoptosis concomitant with attenuated neoplastic proliferation [75], thereby positioning OV-radiotherapy as a compelling candidate for prospective clinical translation.

Some studies have also considered combining OVs with inhibitors of antiviral pathways, which enhance viral replication in cancer cells. The rationale sounds problematic, since the basis for safety and the selective mechanism of action of OVs is precisely that these viruses are more vulnerable to antiviral pathways than pathogenic viruses; therefore, it seems logical that this combination would reduce the safety of the treatment. However, several preclinical studies have found no increased toxicities. This combination has also been applied to PDAC [76], therefore it could deserve further investigation.

The chief combination therapy and the one with the strongest rationale is with other immunotherapeutic agents, such as immune checkpoint inhibitors, cellular immunotherapy, adoptive cellular immunotherapy, cytokines and bi- or tri-specific T cell engagers [77].

The rationale for this combination is that while immunotherapeutic agents fail due to the absence of lymphocytes in the tumor microenvironment, OV replication enhances the recruitment of T cells, which can be subsequently activated by ICIs. The best strategy could envision using OVs that encode chemokines that recruit T cells or other cytokines that synergize with ICIs to achieve T lymphocyte activation [78,79].

Among immune checkpoint inhibitors, anti-PD-1 agents are prominent due to the central role of immune checkpoints in suppressing immune responses and promoting self-tolerance by modulating T-cell activity [80]. Although ICIs targeting PD-1 and its ligand PD-L1 have been widely employed in treating numerous solid tumors, fewer than 1% of pancreatic cancer patients respond to these monotherapies [81]. Thus, combining oncolytic virotherapy with ICIs represents a rational strategy to overcome resistant tumors. Indeed, the pairing of anti-PD-1 with an oncolytic measles vaccine strain was evaluated in an immunocompetent, transplantable PDAC mouse model, revealing increased immune cell infiltration into the TME, enhanced immune activation, and significantly prolonged survival relative to monotherapies—paving the way for future clinical investigations [82].

On the same general theme, it is possible to combine OVs with chimeric antigen receptor (CAR) T cells, with the idea of enhancing the recruitment and the persistence of CAR T cells in the TME. Particularly, OVs induce a complex remodeling of TME, inducing the activation of specific immunogenic cell death pathways, that provide danger signals that can contrast tumor immunosuppression and the production of immune-stimulating molecules, which boost CAR-T functionality [10].

A combination of oncolytic viruses and CAR-T cells was evaluated in subcutaneous human pancreatic xenograft tumor models treated with CF33, an engineered OV expressing a non-signaling truncated CD19 antigen, alongside CAR-T cells. This combinatorial therapy induced significant tumor regression compared to both control and monotherapy groups, suggesting that OVs can convert the cold, immunosuppressive PDAC microenvironment into a “hot” tumor, thereby enabling effective CAR-T targeting [83].

Overall, oncolytic virotherapy holds substantial promise as a novel therapeutic modality for various solid malignancies, including PDAC, harnessing the capacity of OVs to reprogram immunologically quiescent (“cold”) tumor microenvironments into inflamed (“hot”) ones. Notwithstanding this potential, therapeutic efficacy remains markedly patient-dependent. For this reason, these insights underscore the importance of combination therapies for the further advancement of oncolytic virotherapy.

In conclusion, in our opinion OVs have a strong therapeutic potential in the treatment of poorly immunogenic tumors, such as PDAC. Prior clinical studies indicate an outstanding safety profile of this type of treatment and some therapeutic efficacy. However, OVs are still awaiting a “decisive” moment in which they prove that they can be a real game changer in the treatment of cancer. It is important to remember that a lot of completely different vectors can be classified under the “OV” label, and that the therapeutic strategy can vary widely. Therefore, the selection of the vector, of the encoded therapeutic genes, the best delivery strategy, and the combination therapies will be essential for the future of oncolytic virotherapy against PDAC.

## Figures and Tables

**Table 1:** OVs recently tested in clinical trials for PDAC treatment.

Virus	Therapeutic Genes	Other Treatments in Combination	Route of Administration	Clinical Trial Phase
ONYX-015 (Adenovirus)	//	//	Intratumoral	I
ONYX-015 (Adenovirus)	//	Gemcitabine	Intratumoral	I/II
Ad5-DS (Adenovirus)	SR39 herpes simplex virus thymidine kinase fusion/yCD/*mut*TK_SR39_ gene + ADP genes	Gemcitabine	Intratumoral	I
Ad5-yCD/*mut*TK_SR39_*rep*-hIL-12 (Adenovirus)	yCD/*mut*TK_SR39_ + hIL12	5-Fluorouracil + FOLFIRINOX/Gemcitabine/Albumin-bound paclitaxel	Intratumoral	I
VCN-01 (Adenovirus)	PH20	Gemcitabine/Nab-paclitaxel plus Gemcitabine	Intratumoral	I
VCN-01 (Adenovirus)	PH20	Nab-paclitaxel plus Gemcitabine	Intravenous	I
LoAd703 (Adenovirus)	TMZ-CD40L + 4-1BBL	Gemcitabine + Nab-paclitaxel +/− Atezolizumab	Intratumoral	I/II
HF10 (Herpesvirus)	//	//	Intratumoral	I
HF10 (Herpesvirus)	//	Gemcitabine + Nab-paclitaxel/TS-1	Intratumoral	I
OH2 (Herpesvirus)	GM-CSF	//	Intratumoral	Ib/II
VG161 (Herpesvirus)	hIL-12 + hIL-15 + PDL1B	Nivolumab	Intratumoral	I/II
T-VEC (Herpesvirus)	GM-CSF	//	Intratumoral	I
hV01 (Vaccinia virus)	hIL-21	//	Intratumoral	IIa
hV01 (Vaccinia virus)	hIL-21	+/− Tislelizumab	Intratumoral	//
H-1PV (Parvovirus)	//	//	Intravenous	II
Pelareorep (Reovirus)	//	Gemcitabine	Intravenous	IIb
Pelareorep (Reovirus)	//	Paclitaxel/Carboplatin	Intravenous	II
Pelareorep (Reovirus)	//	Pembrolizumab + 5-Fluorouracil/Gemcitabine/Irinotecan	Intravenous	Ib
Pelareorep (Reovirus)	//	Pembrolizumab	Intravenous	II

## Data Availability

No new data were created or analyzed in this study.

## Abbreviations

The following abbreviations are used in this manuscript:

4-1BBL	4-1BB Ligand
Ad5-DS	Ad5-yCD/mutTK(SR39)rep-ADP
AdVs	Adenoviruses
BCM	Baylor College of Medicine
BSLMC	Baylor St. Luke’s Medical Center
CAFs	Cancer-Associated Fibroblasts
CAR-T	Chimeric Antigen Receptor T-cells
CD40L	CD40 Ligand
CTLA-4	Cytotoxic T-Lymphocyte-Associated Protein 4
CXCL1	C-X-C Motif Chemokine Ligand-1
CXCL5	C-X-C Motif Chemokine Ligand-5
CXCL7	C-X-C Motif Chemokine Ligand-7
CXCL8	C-X-C Motif Chemokine Ligand-8
DAMPs	Damage-Associated Molecular Patterns
DCs	Dendritic Cells
GM-CSF	Granulocyte Macrophage Colony-Stimulating Factor
HA	Hyaluronic Acid
hIL-12	Human Interleukin-12
hIL-15	Human Interleukin-15
hIL-21	Human Interleukin-21
HSV	Herpes Simplex Virus
HSV-1	Herpes Simplex Virus type 1
HSV-2	Herpes Simplex Virus type 2
ICD	Immunogenic Cell Death
ICIs	Immune Checkpoint Inhibitors
IL-6	Interleukin-6
IL-10	Interleukin-10
K-Ras	Kirsten Rat Sarcoma Virus
MDSC	Myeloid Derived Suppressor Cells
MSCs	Mesenchymal Stromal Cells
NKs	Natural Killer Cells
oAdVs	Oncolytic Adenoviruses
Ovs	Oncolytic Viruses
OX40L	OX40 Ligand
PAMPs	Pathogen-Associated Molecular Patterns
PDAC	Pancreatic Ductal Adenocarcinoma
PD-1	Programmed Cell Death-1
PD-L1	Programmed Death Ligand-1
PD-L2	Programmed Death Ligand-2
PGE2	Prostaglandin E2
PH20	Human Sperm Hyaluronidase PH20
PSCs	Pancreatic Stellate Cells
SITC	Society for Immunotherapy of Cancer
ssDNA	Single-Strand DNA
T3D	Type 3 Dearing
TAA	Tumor-Associated Antigens
TAMs	Tumor Associated Macrophages
TANs	Tumor Associated Neutrophils
TK	Thymidine Kinase
TME	Tumor Microenvironment
TMZ	Temozolomide
T-reg	Regulatory T Cells
T-VEC	Talimogene Laherparepvec
VACV	Vaccinia Virus
WR	Western Reserve
yCD	Yeast Cytosine Deaminase
